# Enhancing mutation detection in multiple myeloma with an error-corrected ultra-sensitive NGS assay without plasma cell enrichment

**DOI:** 10.1186/s12935-024-03470-7

**Published:** 2024-08-12

**Authors:** Jin Ju Kim, Soo-Jeong Kim, Seoyoung Lim, Seung-Tae Lee, Jong Rak Choi, Saeam Shin, Doh Yu Hwang

**Affiliations:** 1 Department of Laboratory Medicine, Yonsei University College of Medicine, Yongin Severance Hospital, Yongin, Korea; 2 Department of Internal Medicine, Division of Hemato-Oncology, Yonsei University College of Medicine, Yongin Severance Hospital, Yongin, Korea; 3 Graduate School of Medical Science, Brain Korea PLUS Project, Yonsei University College of Medicine, Seoul, Republic of Korea; 4 Department of Laboratory Medicine, Yonsei University College of Medicine, Severance Hospital, 50-1 Yonsei-ro, Seodaemun-gu, Seoul, 03722 Korea; 5 Dxome, Seoul, Republic of Korea; 6 Department of Internal Medicine, Division of Hematology, Yonsei University College of Medicine, Yongin Severance Hospital, 50-1 Yonsei-ro, Seodaemun-gu, Seoul, 03722 Republic of Korea

**Keywords:** Multiple myeloma, Error-corrected next-generation sequencing, Circulating tumor DNA, Plasma cell enrichment

## Abstract

**Background:**

Risk stratification in multiple myeloma (MM) patients is crucial, and molecular genetic studies play a significant role in achieving this objective. Enrichment of plasma cells for next-generation sequencing (NGS) analysis has been employed to enhance detection sensitivity. However, these methods often come with limitations, such as high costs and low throughput. In this study, we explore the use of an error-corrected ultrasensitive NGS assay called positional indexing sequencing (PiSeq-MM). This assay can detect somatic mutations in MM patients without relying on plasma cell enrichment.

**Method:**

Diagnostic bone marrow aspirates (BMAs) and blood samples from 14 MM patients were used for exploratory and validation sets.

**Results:**

PiSeq-MM successfully detected somatic mutations in all BMAs, outperforming conventional NGS using plasma cells. It also identified 38 low-frequency mutations that were missed by conventional NGS, enhancing detection sensitivity below the 5% analytical threshold. When tested in an actual clinical environment, plasma cell enrichment failed in most BMAs (14/16), but the PiSeq-MM enabled mutation detection in all BMAs. There was concordance between PiSeq-MM using BMAs and ctDNA analysis in paired blood samples.

**Conclusion:**

This research provides valuable insights into the genetic landscape of MM and highlights the advantages of error-corrected NGS for detecting low-frequency mutations. Although the current standard method for mutation analysis is plasma cell-enriched BMAs, total BMA or ctDNA testing with error correction is a viable alternative when plasma cell enrichment is not feasible.

**Supplementary Information:**

The online version contains supplementary material available at 10.1186/s12935-024-03470-7.

## Introduction

Multiple myeloma (MM), is a plasma cell neoplasm that predominantly affects elderly individuals and accounts for 10% of hematologic neoplasms [1]. The initial choice of therapy for patients with plasma cell neoplasms is based on clinical criteria; however, identifying cytogenetic abnormalities in plasma cells is valuable for risk stratification [2]. Several somatic driver mutations, such as *KRAS*,* NRAS*, and *TP53*, are related to MM [3, 4]. Therefore, many molecular genetic studies, including chromosomal analysis, interphase fluorescence in situ hybridization (FISH) and next-generation sequencing (NGS), are performed on MM patients.

Bone marrow aspirates (BMAs) from MM patients are mixture of normal hematopoietic cells and malignant plasma cells. The composition fraction of plasma cells varies from 10% to > 80%, so it is crucial to detect the genetic abnormality of malignant plasma cells, which can be diluted by normal cells. To increase the analytical sensitivity of FISH or NGS to detect molecular abnormalities, plasma cell enrichment techniques such as fluorescence immunophenotyping and interphase cytogenetics as a tool for the investigation of neoplasms (FICTION), fluorescence-activated cell sorter (FACS), or magnetic-activated cell sorting (MACS) are used in laboratories [5–7]. However, the plasma cell enrichment process has some disadvantages, such as the associated cost (for equipment, reagents, and labor), time (particularly for the cell sorting step), technician training, and the need for large amounts of fresh samples [7]. Due to these drawbacks, the application of enrichment techniques in routine clinical practice is limited.

There are frequent errors in NGS during processing due to DNA damage and sequencing steps. These errors create barriers to sensitive mutation detection. Therefore, several error correction strategies have recently emerged in the clinical NGS field to detect low-allele frequency mutations for circulating tumor DNA (ctDNA) or measurable residual disease (MRD) analysis [8, 9]. Error correction strategies, such as molecular barcoding or in silico error suppression, can increase the detection capability of NGS to below 1% of variant allele frequencies (VAF) [10–13]. We developed a positional indexing sequencing (PiSeq) analysis method that tags the beginning and end parts of DNA molecules. By recognizing sequencing reads with the same start and end points as a group, the method is able to distinguish and correct errors in sequencing [14, 15].

**Fig. 1:**
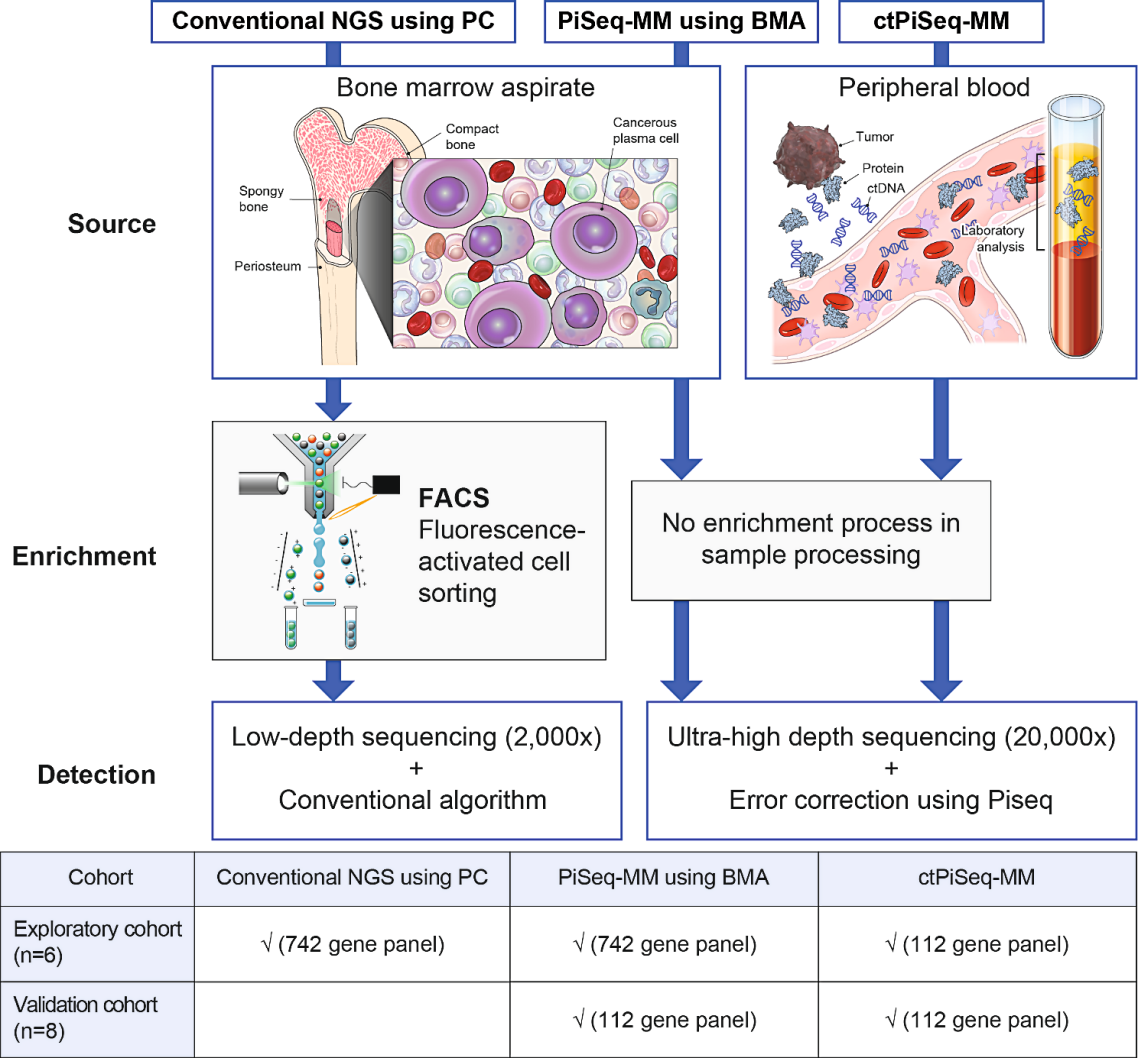
Overview of the study design for validation of an ultrasensitive NGS assay An illustration of the study design

This study evaluated whether mutation detection sensitivity could be increased in MM patients using our error-corrected ultrasensitive NGS assay (PiSeq-MM) without plasma cell enrichment. We hypothesized that an error-corrected algorithm would enable us to detect somatic mutations in BMA without the need for plasma cell enrichment, similar to blood sample analysis. Using total cells for NGS can streamline the clinical workflow by eliminating the need for cell enrichment, and thereby reduce time and effort. Additionally, we conducted NGS on matched blood samples to investigate whether ctDNA analysis can infer somatic mutations in malignant plasma cells. An overview of this study is depicted in Fig. 1.

## Materials and methods

### Study samples

Diagnostic BMAs and matching blood samples from 14 patients who visited the Yongin Severance Hospital between March 2020 and March 2023 were included. Six patients were included in the exploratory cohort and eight in the validation cohort. Patients in the exploratory cohort had NGS results from fresh BMAs with a plasma cell enrichment step performed using our institute diagnostic panels (conventional NGS using PC). The panel included 742 target genes with therapeutic, prognostic, and diagnostic properties in a variety of cancers, including lymphomas and myeloma (Supplementary Table S1). The plasma cell enrichment process was successful in five out of six patients’ samples and the samples are proceeded to NGS analysis. In the sample in which the enrichment step failed (P3), the total DNA from the BMA was used for NGS analysis. General NGS strategies were applied without error-corrected bioinformatics algorithms with a mean sequencing depth of 522×. In this case, an analytical sensitivity of 5% was assumed.

The patients were pathologically diagnosed with MM according to the 2014 International Myeloma Working Group (IMWG) criteria [16]. The following clinical data were collected from the electronic medical records: age, sex, test results (including cytogenetics), bone marrow study, and clonality test results. The baseline clinical features of the 14 MM patients whose clinical samples were used in this validation are summarized in Table 1.

**Table 1:** Clinicopathologic characteristics of the patients in this study

	Patients (*N* = 14)	
	Exploratory set (*N* = 6)	Validation set (*N* = 8)
Age, years (median, range)	71 (42–84)	60 (56–75)
Hemoglobin, g/dL (median, range)	9.45 (6.7–11.3)	7.95 (5.8–11.7)
Bone marrow plasma cells (median, range)	73.9% (49.4 − 82.8%)	38.5% (11.3 − 91.4%)
Serum M protein, g/dL (median, range)	4.37 (0.92–5.72)	2.06 (0.08–5.59)
Type (n,%)		
IgG κ	3 (50.0%)	3 (37.5%)
IgA κ	1 (16.7%)	-
IgG λ	1 (16.7%)	1 (12.5%)
IgA λ	-	1 (12.5%)
κ light chain disease	-	2 (25.0%)
λ light chain disease	1 (16.7%)	1 (12.5%)
Karyotype (n,%)		
Hyperdiploidy	2 (33.3%)	1 (12.5%)
Complex karyotype	3 (50.0%)	1 (12.5%)
Translocation	-	1* (12.5%)
Deletion 17p	-	-
Normal karyotype	1 (16.7%)	5 (62.5%)
FISH (n,%)		
t(4;14)	1 (16.7%)	-
t(11;14)	1 (16.7%)	2 (25.0%)
t(14;16)	-	-
t(14;20)	-	-
Elevated LDH	1 (16.7%)	2 (25.0%)
Elevated β2-microglobulin	6 (100.0%)	8 (100.0%)
IgH/K clonality positivity	6 (100.0%)	8 (100.0%)

*46,XY, t(8;14)(q24.1;q32),t(11;14)(q13;q32)

### Sample preparation

Fresh BMAs from exploratory cohorts collected in ethylene diamine tetra-acetic acid (EDTA) tubes were used for conventional NGS using PC. Plasma cell enrichment was performed as follows: buffy coats were isolated from BMAs and diluted with erythrocyte lysis buffer. The mix was incubated at room temperature for 20 min, and then centrifuged at 2100 rpm for 5 min. The cell pellet was resuspended in phosphate-buffered saline. After three washing cycles, the concentration was adjusted to 1 × 10⁶ − 4 × 10⁷ cells/mL. Antibody staining was performed using anti-CD38-FITC and anti-CD138-PE (Beckman Coulter, CA, USA). Then, plasma cell sorting was conducted on a BD FACS Melody™ (BD Biosciences, San Jose, CA, USA) or S3e™ Cell Sorter (Bio-Rad Laboratories, Hercules, CA, USA).

For ctDNA analysis, blood samples were obtained from patients at the time of diagnosis. Twenty milliliters of whole blood in a DxTube (Dxome, Seoul, Republic of Korea) was used. The samples were processed within 4 h at a constant temperature of 4 °C. Plasma was isolated by double centrifugation (1900 × g for 15 min). Peripheral blood mononuclear cells (PBMCs) were transferred to fresh tubes in 1 ml aliquots. Supernatants were also separately aliquoted in fresh tubes. Frozen aliquots of plasma were stored at -80 °C until ctDNA extraction. The ctDNA was extracted from 4 mL of plasma using magnetic circulating DNA Maxi Reagent (Dxome) according to the manufacturer’s instructions.

EDTA-BMAs from all 14 patients were also double centrifuged. Buffy coats were collected and then frozen in aliquots at -80 °C. Genomic DNA (gDNA) from PBMCs and BMA buffy coats was extracted using the QIAsymphony DNA Mini Kit (Qiagen, Hilden, Germany) according to the manufacturer’s guidelines. Library preparation was performed using 2.5–30 ng of ctDNA and 110–200 ng of sheared gDNA using the DxSeq Library prep reagent (Dxome). For each sample, PBMCs were sequenced as germline-matched controls using identical panel and library kits targeting an average depth of > 2,500×. The pooled libraries were paired-end sequenced (2 × 150 bp) on the NovaSeq 6000 System (Illumina, San Diego, CA, USA). Bioinformatics pipelines used for the analysis of NGS data consist of multiple steps, such as demultiplexing, read alignment, deduplication, base calibration and variant calling. An additional variant calling step with our error-correction pipeline, the PiSeq algorithm (Dxome), was used to differentiate low-frequency mutations from amplification artifacts and sequencing errors by calculating the genomic positions of mapped reads [14]. Variants were annotated using DxSeq software (Dxome) with public database information. Identified variants were visually confirmed with Integrative Genome Viewer (Broad Institute, Cambridge, MA, USA). Genic copy number variants (CNVs) and CNVs at the whole genome level were analyzed using DxSeq software (Dxome, Sungnam, South Korea). Germline variants were removed using parallel NGS data from PBMC-derived DNA.

### Statistical analysis

Statistical analyses were performed using MedCalc version 18.2.1 (MedCalc Software; Mariakerke, Belgium). For continuous data, the Shapiro–Wilk test was used to detect departures from normality. Variables were compared using the Mann–Whitney *U* test. The Passing–Bablock regression was used to compare the VAF between samples. The Spearman rank correlation coefficient (r) was calculated. Statistical significance was defined as *p* < 0.05.

## Results

### Ultra-high depth ngs sequencing with the piseq algorithm: enhancing mutation detection in MM without plasma cell enrichment

Our primary objective was to determine whether ultrahigh-depth NGS sequencing with the Piseq algorithm could effectively detect meaningful variants in MM without the need for plasma cell enrichment (PiSeq-MM). To achieve this, we conducted NGS using BMA samples without performing the enrichment step. Our study cohort consisted of six MM patients who had previously undergone conventional NGS using PC.

For the analysis, we used the same targeted NGS panel of 742 genes. The median sequencing depth of PiSeq-MM using the six BMAs was 14,427×. In the comparison, mutations were detected in five out of six conventional NGS using PCs (83.3%), while all six PiSeq-MM using BMAs had detectable mutations (Supplemental Table S2).

**Fig. 2:**
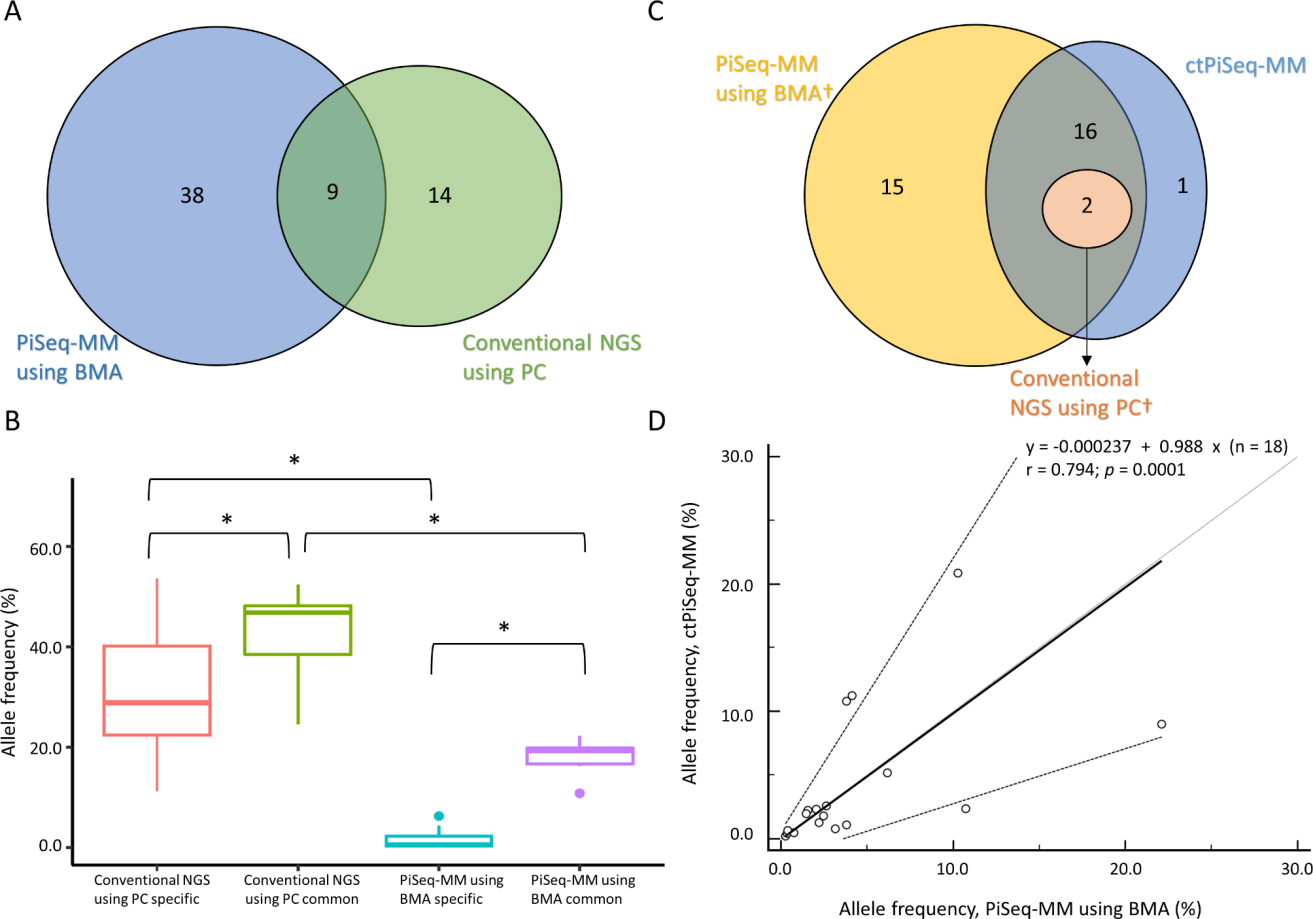
Concordance analysis of SNV/indel mutations detected in samples from the exploratory cohort. **(A)** Venn diagram showing the number of mutations detected in PiSeq-MM using BMA and conventional NGS using PC. **(B)** VAF distribution of mutations identified by conventional NGS using PC specific, shared by both conventional NGS using PC and PiSeq-MM using BMA, and by PiSeq-MM using BMA specific. **(C)** Venn diagram of mutations shared by PiSeq-MM using BMA, conventional NGS using PC and ctPiSeq-MM. Only targeted genes shared between each NGS panels were considered. **(D)** Correlation of VAFs between the two NGS panels. *p < 0.05 †Targeted genes shared by both NGS panels, 104 out of 742 genes are considered

The total number of somatic mutations detected in conventional NGS using PCs ranged from 0 to 7 mutations per patient, amounting to 23 mutations in total. In contrast, PiSeq-MM using BMAs identified 47 somatic mutations, with a range of 2 to 16 mutations per patient. Notably, 39.1% (*n* = 9/23) of the mutations detected in PiSeq-MM using BMAs also showed a median VAF of 46.8% in the plasma cell-enriched samples (Fig. 2A and B). Mutations that were not discovered in PiSeq-MM using BMAs had a low representation in conventional NGS using PCs, with a median VAF of 28.8% (Fig. 2B). None of the 14 non-overlapping mutations are known to impact the clinical diagnostic outcome.

PiSeq-MM using BMAs uncovered an additional 38 somatic mutations that were not detectable in conventional NGS using PCs. Among these, there were 7 variants affecting *KRAS* [17, 18] and 6 variants affecting *NRAS* [18], both of which are known driver mutations in MM (Supplemental Table S2). The VAFs of these 38 somatic mutations ranged from 0.1 to 10.3% with a median VAF of 0.5% (Fig. 2B), which is near or below the analytical sensitivity of general NGS, 5%.

Importantly, there was no correlation between the VAF of conventional NGS using PCs and PiSeq-MM using BMA (*r* = 0.367, *p* = 0.3317). This finding indicates that the mutation detection with PiSeq-MM using BMA is not solely dependent on the VAF observed in plasma cell-enriched samples.

### Exploring the potential of ctDNA analysis in MM: concordance and mutational landscape compared to conventional NGS

To investigate the potential of ctDNA in MM, we developed a targeted NGS panel comprising a smaller number of genes (112 genes) than were included in the comprehensive panel (742 genes) (Supplementary Table S1). The selected 112 genes were chosen based on the following criteria: (a) commonly found in myeloma patients tested with our institute’s conventional NGS panel (742 genes), (b) involved in important signaling pathways in multiple myeloma e.g. the MAPK, MYC, DNA repair and NFKB pathways, and (c) treatment targets or candidates for drug resistance in multiple myeloma (e.g. *IKZF3*,* BCL2*,* PTEN* and *NFKB2*) [19–22]. Additionally, to ensure that the ctDNA NGS panel can be used for patients with both myeloma and lymphoma, genes found in non-Hodgkin lymphoma (e.g., *CD7*) were also included. This selection of these genes was meticulously curated based on an extensive review of relevant literature, databases, and guidelines by a team of expert medical oncologists.

We conducted ctDNA analysis using the targeted NGS panel comprising 112 genes and employed the Piseq algorithm (ctPiSeq-MM) on matched blood samples from six patients in an exploratory cohort. The median sequencing depth of ctPiSeq-MM was 68,048×. A total of 19 somatic mutations were detected across the six ctPiSeq-MM samples, ranging from 0 to 6 mutations per patient.

Considering only the mutations present in the genes shared between the two NGS panels (Supplementary Table S1), we found that all mutations identified in conventional NGS using PC, specifically *NRAS* Q61R and *NRAS* G13D, were consistently detected in both Piseq-MM using BMAs and ctPiSeq-MM analyses. Almost all mutations (94.7%, 18/19) were detected in ctPiSeq-MM, except one mutation (*KRAS* G12S) with a very low VAF of 0.24% (Fig. 2C, Supplementary Table S2). Furthermore, there was a substantial correlation between the VAF of Piseq-MM using BMAs and ctPiSeq-MM (*r* = 0.794, *p* = 0.0001; Fig. 2D), indicating a strong concordance between the mutation profiles obtained from both methods.

Based on our initial observations in a small exploratory test cohort, we proceeded to validate our findings in a separate cohort of eight MM patients to assess the concordance of detected mutations between gDNA from BMA and ctDNA using the 112 gene NGS panel. For this validation cohort, the median sequencing depths of PiSeq-MM using BMAs and ctPiseq-MM were 60,444× and 78,862×, respectively.

We identified a total of 78 somatic mutations from PiSeq-MM using BMAs and 45 somatic mutations from ctPiseq-MM (Supplementary Table S3). The median VAF of somatic mutations detected in PiSeq-MM using BMAs was 0.15% (range: 0.04 − 9.58%), while the median VAF of mutations in ctPiSeq-MM was 0.96% (range: 0.09 − 21.19%).

**Fig. 3:**
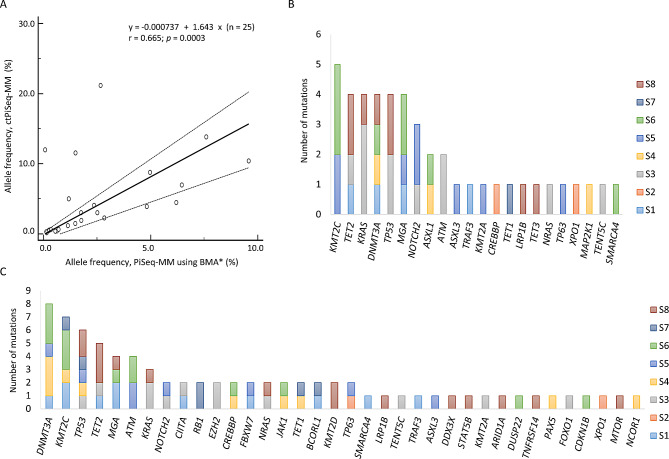
**(A)** Correlation of SNV/indel allele frequency between PiSeq-MM using BMA and ctPiSeq-MM with a 112 gene targeted panel in the validation cohort. Mutation spectrum of **(B)** ctPiSeq-MM and  **(C)** Piseq-MM using BMAs in the validation cohort

Among the mutations detected, 25 mutations were shared in both PiSeq-MM using BMAs and ctPiSeq-MM of matched patients. There was a moderate degree of correlation between the VAF of PiSeq-MM using BMAs and ctPiSeq-MMs (*r* = 0.665, *p* = 0.0003; Fig. 3A). These results suggest a reasonable concordance in mutation detection between the two sample types, further supporting the potential utility of ctDNA analysis in MM.

In all eight patients, at least one mutation was detected in both the BMA and blood samples. On average, PiSeq-MM using BMAs identified 11 mutations (range: 2–16), whereas ctPiSeq-MMs detected 6 mutations (range: 1–11). Interestingly, the most frequently mutated gene in the blood samples was *KMT2C* (Fig. 3B**)**. In contrast, the most frequently mutated gene detected in PiSeq-MM using BMAs was *DNMT3A*, followed by *KMT2C*,* TP53*,* MGA*,* ATM*, and *KRAS* (Fig. 3C). These findings provide valuable insights into the mutational landscape of MM, and highlight differences in mutation frequencies between the two sample types.

In one patient (S4), a CNV was detected, specifically a partial *KIT* gene deletion involving deletion of exons 8–21. Notably, this CNV was detected only in PiSeq-MM using BMAs and not in ctPiSeq-MMs.

### Chromosomal abnormalities and detection challenges in MM patients: insights from multiple NGS protocols

**Fig. 4:**
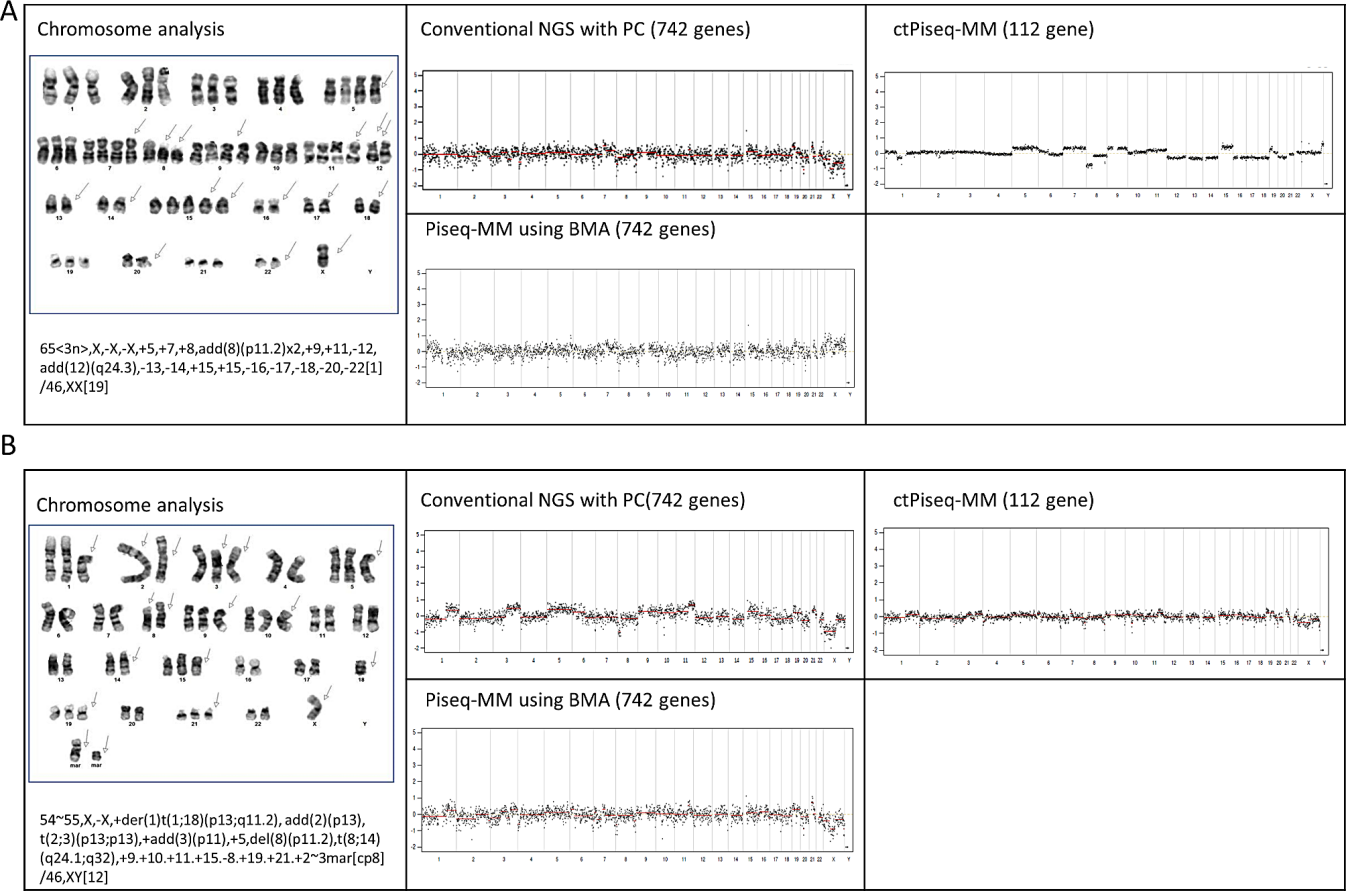
Chromosome analysis results by test method in patients with hyperdiploidy: **(A)** P1 and **(B)** P6

In the exploratory set, a majority of patients (83.3%, 5/6) exhibited chromosomal structural abnormalities as determined by cytogenetic analysis. Specifically, two patients (P1 and P6) showed hyperdiploidy, while three patients (P2, P3, and P5) had complex karyotypes. Notably, cases displaying hyperdiploidy in karyotyping also had identifiable chromosomal abnormalities in conventional NGS using PC, PiSeq-MM using BMA, and ctPiseq-MM. However, it is essential to acknowledge that the chromosomal abnormality results using NGS may not be entirely consistent with karyotyping (Fig. 4).

In contrast, patients with complex karyotypes or normal karyotypes sometimes exhibited abnormalities in conventional NGS using PC or Piseq-MM with BMA. However, interestingly, ctPiseq-MM did not show abnormalities in cases with normal karyotypes. This finding reveals the limitations of whole gene CNV analysis when using blood samples (Supplementary Fig. 1). Additionally, even when employing the Piseq algorithm in the samples from the validation cohort, it was challenging to detect chromosome abnormalities using whole gene CNV analysis (Supplementary Fig. 2).

### Implementation of the Piseq Algorithm for NGS analysis in MM patients

Based on the insights gained from this study, we adopted the Piseq algorithm for BMAs from MM patients undergoing NGS, commencing in March 2022. NGS testing was initiated using plasma cell-enriched BMAs. In cases where enrichment was unsuccessful, the analysis was conducted on total BMAs. Between March 2022 and July 2023, 16 BMAs were subjected to NGS (Supplementary Table S4). The median arrival time at the laboratory was 41.0 h (range: 39.0–112.3 h), with plasma cell enrichment failing in the majority of cases (14/16, 87.5%).

Despite relying on total BMAs for most cases, mutations were identified in all samples, with potential driver mutations detected in 14 of 16 samples. The median VAF spanned from 0.1 to 46.6%, while the plasma cell burden in BMAs ranged from 10.8 to 99.6%. Notably, the median VAF of mutations and the plasma cell burden in BMAs showed no correlation (*p* = 0.793).

## Discussion

Genetic variants have been linked to drug resistance and prognosis in MM patients [23–25], and NGS tests, cytogenetic studies, and FISH analyses are crucial for comprehensive genetic analysis. Additionally, detecting residual cancer or low-fraction mutations requires very sensitive methods. Enriching plasma cells from bone marrow aspirations is a commonly employed technique to enhance detection sensitivity in FISH analysis and NGS [26]. However, this enrichment step adds labor and technical costs and necessitates timely sample delivery to prevent CD138 shedding [27], which can lead to false negative results. False negatives may also occur in cases where plasma cell neoplasms lack CD138 expression, although this is rare [26]. Other challenges of plasma enrichment in clinical laboratories include the need for relatively large sample amounts, and occasional failure. These limitations show the need for an alternate strategy for profiling unenriched cells (Table 2).

**Table 2:** Comparison of samples for genetic profiling in multiple myeloma patients

Sample type	Pros	Cons
Bone marrow aspirate	Easy sample handling	Low sensitivity due to hemodilution
		Only applicable to cases with bone marrow involvement
Plasma cell enriched sample	Increased detection sensitivity through high tumor fraction	Only applicable to cases with bone marrow involvement
		Low throughput - Time: hands on time, approximately 4 h - Workload: extra specialist required to interpret flow cytometry
		Facility, extra kit expanse for cell sorting
		Requires a fresh sample - At least 9 mL EDTA blood with at least 25,000 plasma cells
		Fail rate, approximately 20%
Samples with an error corrected NGS algorithm	Easy sample handling with add-on algorithm	Low tumor fraction in samples
	No need for STAT preparation of sample or extra requirements for cell enrichment	Requires high analytical sensitivity (~ 1%)

Error-corrected bioinformatics can help to overcome the limitations of the plasma cell enrichment step while still generating compatible mutation analysis data for plasma cell- unenriched total BMA samples. When utilizing samples collected within a 24-hour timeframe, the enrichment process using the MAC method has been reported to exhibit a failure rate ranging between 10 and 22% depending on the plasma cell burden in the bone marrow [28]. Moreover, enrichment failure was observed in 16.7% (1/6) of an exploratory set using fresh BMA samples in this study. However, in clinical samples from actual patients, the failure rate of the enrichment step exceeded expectations, likely due to the extended duration between bone marrow sample aspiration and the enrichment process.

Despite the predominant use of total BMAs, mutations were identified across all samples, with potential driver mutations identified in most cases. In clinical testing with patient specimens, enrichment often encounters failures, potentially exacerbated by prolonged transit time of specimens when samples are referred to other medical facilities. The VAF of mutations was notably low, considering the plasma cell fractions of BMAs. Some mutations were detected only in conventional NGS using PCs, and these mutations tended to have lower VAFs than those detected by PiSeq-MM using BMAs. This discrepancy could arise from reduced plasma cell fractions that include other hematopoietic cells or hemodilution when using total BMA.

Although the average VAF of these mutations was lower than that of mutations detected simultaneously in the total BMA, some mutations had VAFs exceeding 45% (e.g., *IGLL5* M42T (P2) and, *ZFHX4* P3154A (P6)). This difference is thought to be due to variations in clone composition caused by the degree of hemodilution in the BMAs used for the tests. Conventional NGS using PCs was conducted using fresh, first or second pulled BMAs obtained during the aspiration procedure. In cases of PiseqMM using BMAs, frozen aliquots likely included subsequently aspirated samples. As a result, differences in clone burden and composition might exist between the samples [29]. However, in our study, these 14 mutations were not clinically significantly different in terms of diagnostic outcomes. In contrast, the nine mutations detected by both methods had a higher average VAFs compared to those found only by conventional NGS using PCs, including clinically significant *NRAS* gene mutations. Therefore, mutations detected in both tests are likely crucial for the disease, regardless of VAF, and may represent founder mutations that occurred early in clonal evolution and are shared among various clones. In addition, working with total BMA instead of sorted PCs may have disadvantages related to clonality/subclonality determination, our method effectively detected variants with VAF as low as 5%, ensuring variant identification across all samples and detect key mutations in disease development.

However, due to the relatively low VAF and difficulties in CNV analysis at the whole genome level, plasma cell-enriched BMA samples remain optimal for genetic analysis. Therefore, the most accurate option for NGS analysis remains plasma cell-enriched BMA with error-corrected bioinformatics. Nevertheless, if plasma cell enrichment is not feasible due to limited sample size or technical issues, applying error-corrected bioinformatics alone can still detect some informative mutations for risk stratification.

Although BMA is the preferred sample for sequencing, its ability to detect MM clones may be hindered by an inhomogeneous infiltration pattern [30]. In recent years, ctDNA analysis has emerged as an alternative method for tissue genomic DNA analysis and monitoring residual cancers noninvasively in many solid cancers [8, 14]. Our study demonstrated that ctDNA testing with error-corrected bioinformatics not only yielded similar results to bone marrow samples but also detected mutations with low variant frequency.

This study was conducted with a very small number of patient samples. Therefore, it is challenging to interpret the results as representative of the mutation prevalence in the MM patient population. However, the mutations with a high prevalence in larger existing MM cohorts with NGS data such as those in the *KRAS*, *NRAS*, and *TP53* genes were identified in the total BMA (eight validation cohort and fourteen clinical patients whose plasma cell enrichment failed) and blood samples (eight validation cohort) of patients using Piseq algorithm (Supplementary Table S5). This suggests general concordance with the findings of existing studies [3, 31, 32]. However, *DNMT3A* and *KMT2C* mutations were observed at higher frequency in total BMA and blood samples compared to other studies. Kogure et al. [33]. reported the same pattern in ctDNA analysis in relapsed/refractory MM patients, in whom the majority of mutations in clonal hematopoietic (CH)-related genes, such as *DNMT3A* and *TET2* were detected only by ctDNA, in line with our results. CH gene mutations might have originated from nonmalignant hematopoietic cells not only in blood, but also in BMA, resulting in greater detection of CH mutations with very low frequency when using our PiseqMM. However, this should be further researched with a larger cohort to determine whether the detection frequency is indeed higher when applying this method to the MM patient group.

By jointly analyzing and interpreting genetic results from both BMA and ctDNA analyses in MM patients, not only disease monitoring but also information on the mutation spectrum of myeloma burden from sites other than the biopsy can be obtained. This approach proves valuable even in challenging scenarios, such as plasmacytoma or a dry-tapped marrow [34, 35]. Moreover, recent study suggesting risk stratification model using ctDNA mutations in relapsed/refractory patients highlights the possible clinical application of ctDNA in near future [33].

As this was a pilot study for method validation, further research involving a larger number of patients is required in the future. More extensive investigations are also necessary to confirm the association between the obtained results and prognosis. Prospective studies on MM patients, including those with plasmacytoma, will be essential to establish the ctDNA test method’s utility as a comprehensive genetic analysis tool.

## Conclusion

This research provides valuable insights into the genetic landscape of MM and highlights the advantages of error-corrected NGS for detecting low-frequency mutations. The results suggest that PiSeq-MM can effectively detect somatic mutations in MM patients without the need for plasma cell enrichment. ctDNA analysis showed potential utility in identifying somatic mutations in malignant plasma cells. Although the current standard method for mutation analysis is still the use of plasma cell-enriched BMAs, total BMA or ctDNA testing with error correction is a viable alternative when plasma cell enrichment is not feasible.

### Data availability

The data that support the findings of this study are available from the corresponding author, [SS], upon reasonable request.

### Electronic supplementary material

Below is the link to the electronic supplementary material.

Supplementary Material 1

## Abbreviations

MM: Multiple Myeloma
NGS: Next-Generation Sequencing
FISH: Fluorescence In Situ Hybridization
FICTION: Fluorescence Immunophenotyping and Interphase Cytogenetics as a Tool for the Investigation of Neoplasms
FACS: Fluorescence-Activated Cell Sorter
MACS: Magnetic-Activated Cell Sorting
ctDNA: Circulating Tumor DNA
MRD: Measurable Residual Disease
VAF: Variant Allele Frequencies
PiSeq: Positional Indexing Sequencing
BMA: Bone Marrow Aspirate
PBMCs: Peripheral Blood Mononuclear Cells
gDNA: Genomic DNA
CNVs: Copy Number Variants
IMWG: International Myeloma Working Group
