# Supplementary material for: Enhancing mutation detection in multiple myeloma with an error-corrected ultra-sensitive NGS assay without plasma cell enrichment
Source: Cancer Cell Int. 2024 Aug 12;24:282. doi: 10.1186/s12935-024-03470-7 (PMC11318258; doi:10.1186/s12935-024-03470-7)
Supplement: Supplementary file 1 — Supplementary Material 1 [file 12935_2024_3470_MOESM1_ESM.docx]

**Supplementary Figure 1. Chromosome analysis results by test method in exploratory set patients; (A) P2, (B) P3,(C) P4 and (D) P5.**


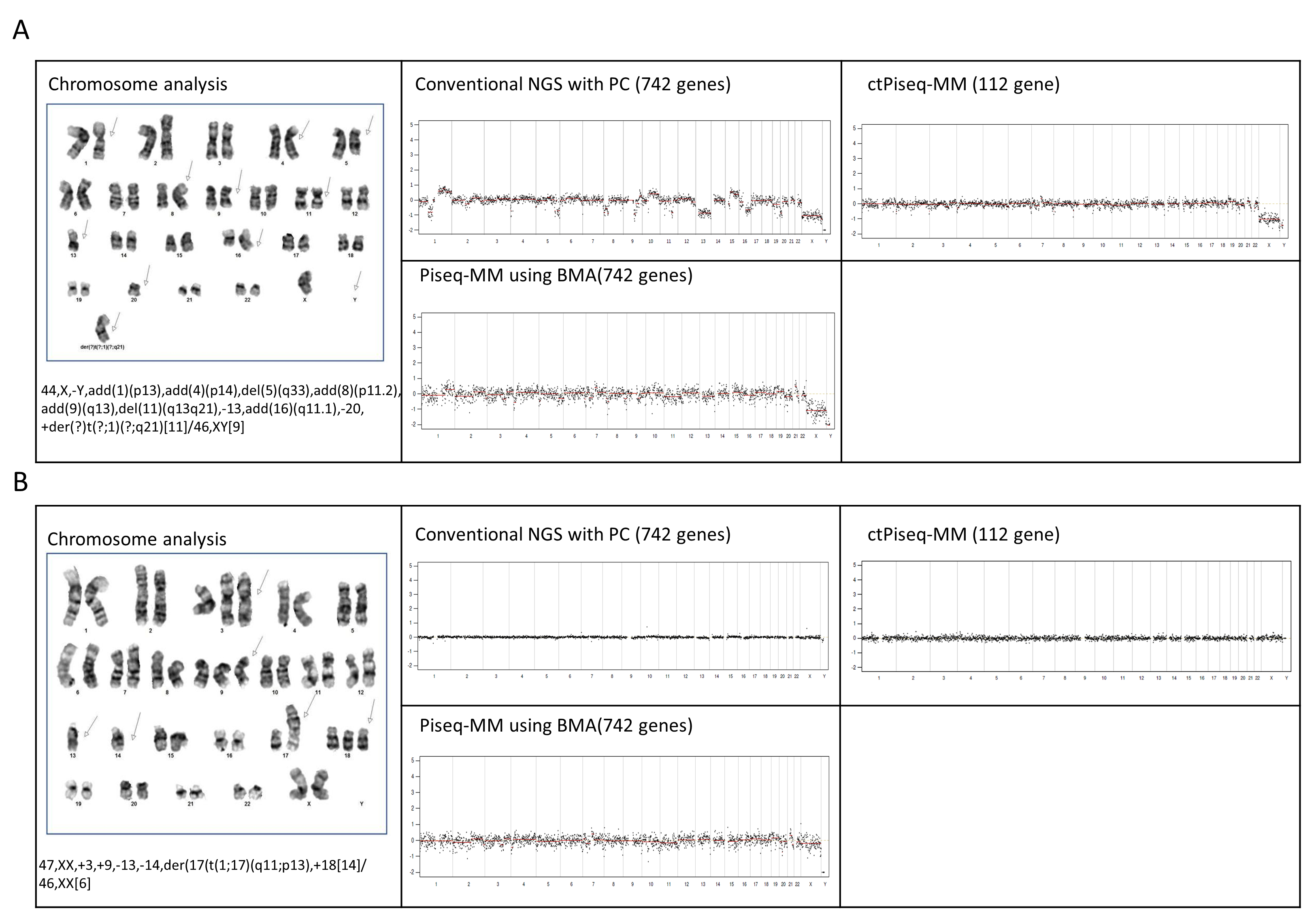

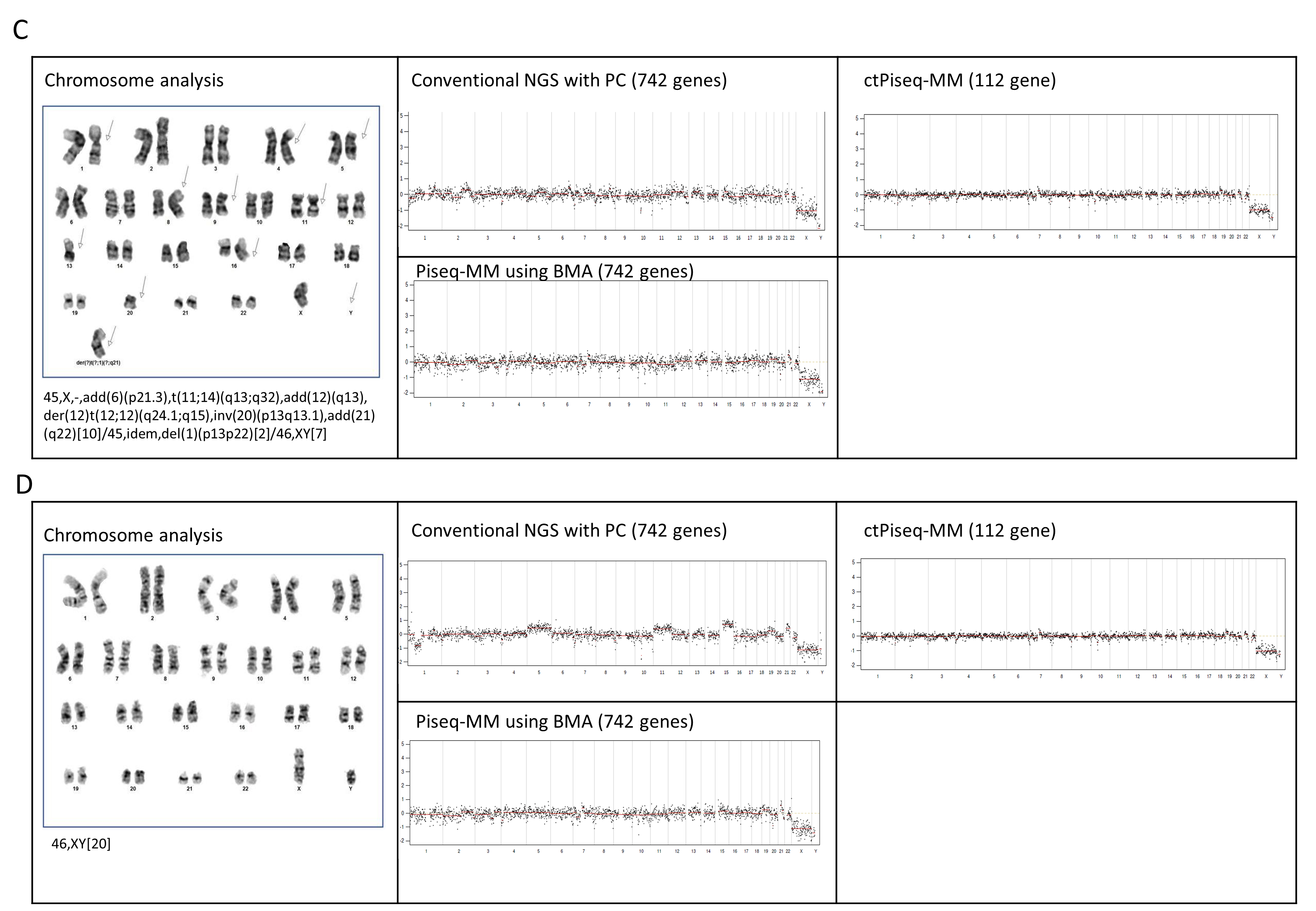


**Supplementary Figure 2. Chromosome analysis results by Piseq-MM using BMA and ctPiseq-MM in validation cohort**


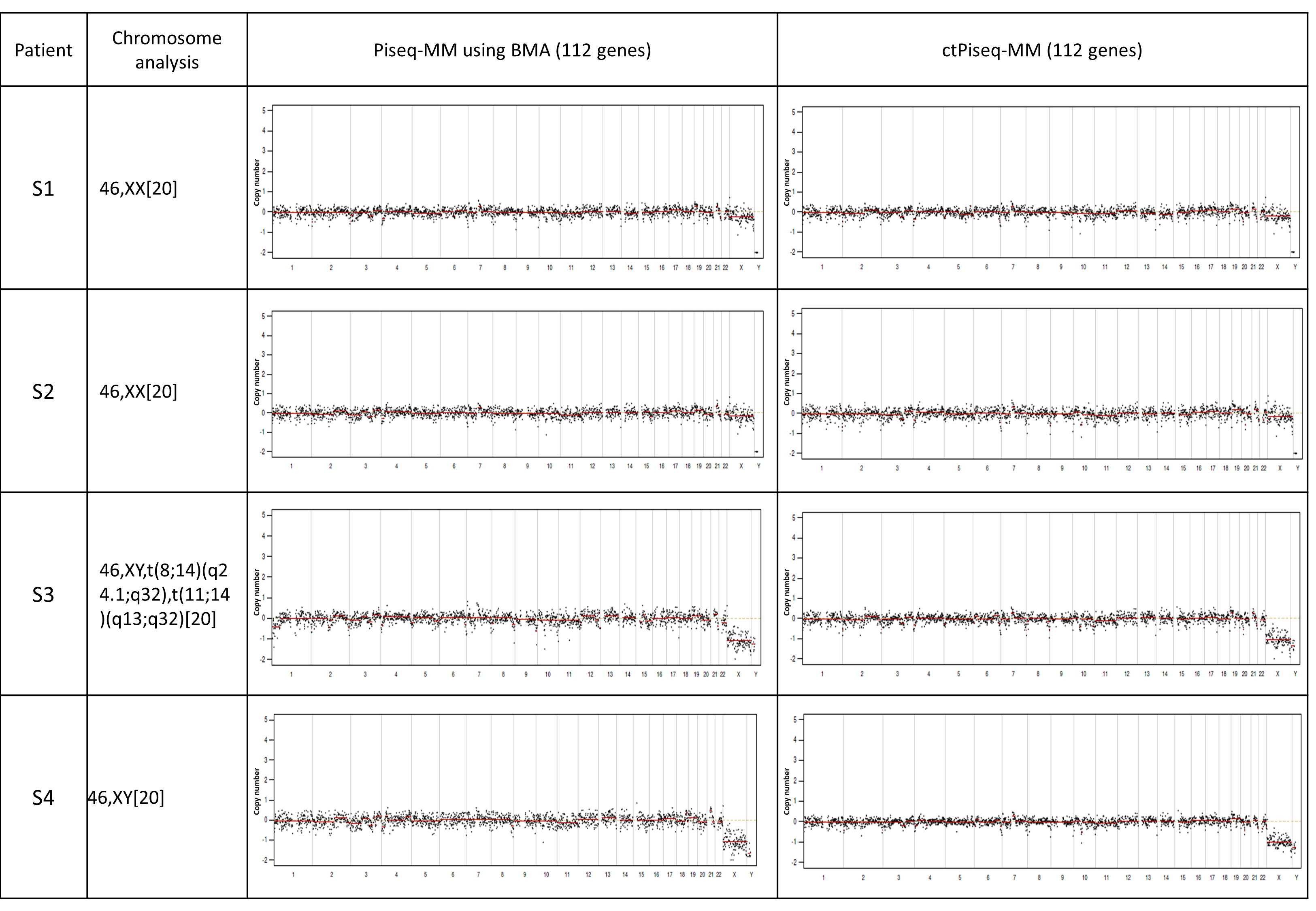

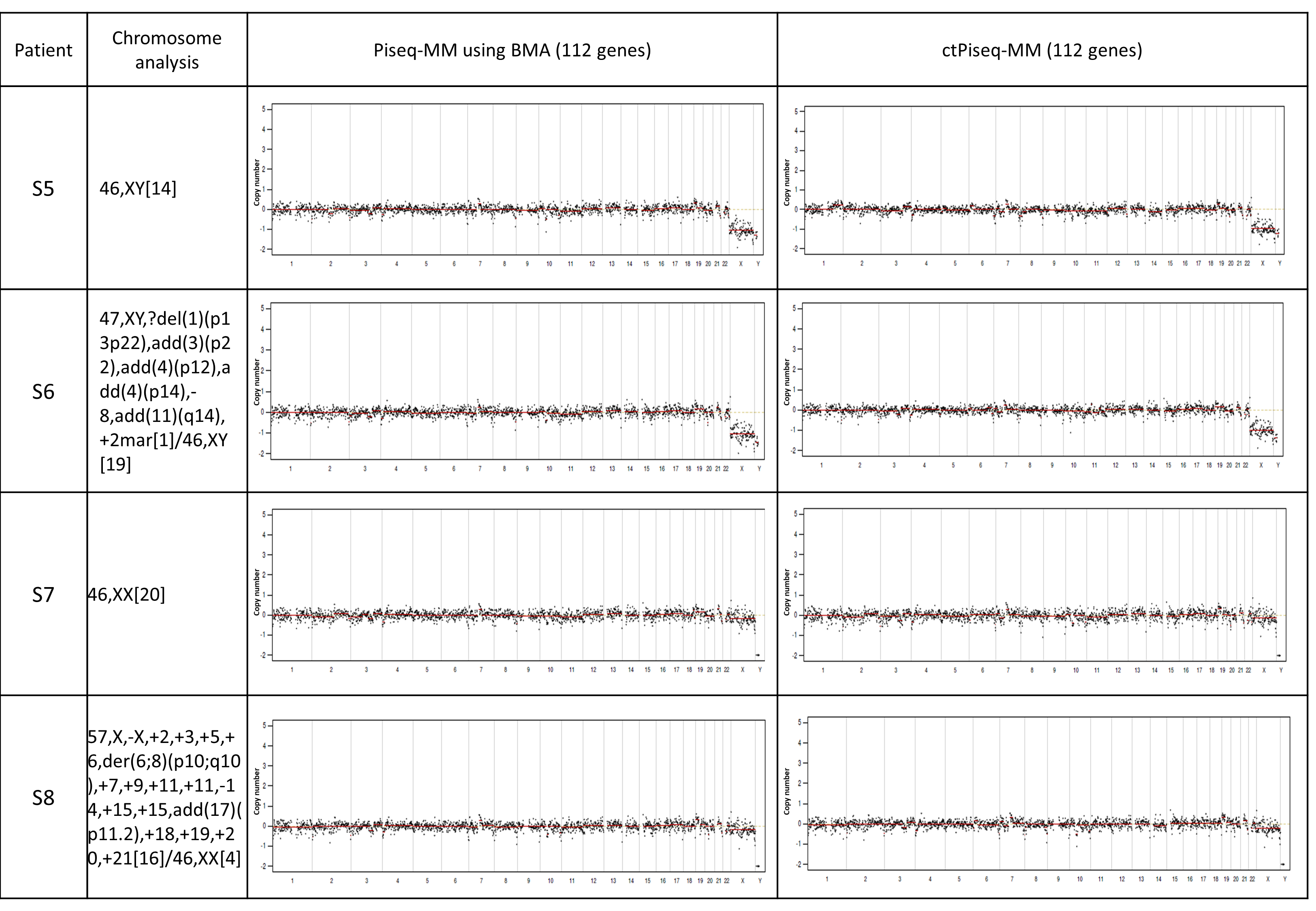


**Supplementary Table S1. List of genes included in the NGS panel (742 genes).**

The text in bold indicates the genes commonly included in the 112 gene targeted NGS panel; *ALK, ASXL3, BIRC6, DUSP22, FYN, NFKB2, PAX5, TET3* genes are only included in 112 gene panel

| **Genes** | | | | | | | | | | | | | | |
| --- | --- | --- | --- | --- | --- | --- | --- | --- | --- | --- | --- | --- | --- | --- |
| *ABCA13* | *BCL10* | *CD81* | *CUL4A* | *EGR2* | *FLYWCH1* | *IFNA14* | ***KRAS*** | *MKLN1* | *NR3C1* | ***PLCG1*** | *RELN* | *SETD1A* | *SYNE1* | *U2AF1* |
| *ABCA3* | ***BCL11B*** | *CD83* | *CUL4B* | *EIF2AK4* | *FMN1* | *IFNA4* | *KRTAP16-1* | *MLH1* | ***NRAS*** | *PLCG2* | *RERE* | ***SETD1B*** | *TAF1* | *U2AF2* |
| *ABCA6* | ***BCL2*** | *CDC123* | *CUL9* | *EIF4A1* | ***FOXO1*** | *IFNGR1* | *LAMA2* | *MLH3* | *NRXN3* | *PLEKHG1* | *RET* | ***SETD2*** | *TBC1D26* | *UBE2A* |
| *ABCA7* | *BCL2L11* | *CDC25C* | ***CXCR4*** | *EIF4A2* | *FOXP1* | *IFNGR2* | *LAMB4* | *MMP8* | *NSD1* | *PLEKHG5* | *REV3L* | *SETD6* | ***TBL1XR1*** | ***UBR5*** |
| *ABCC9* | ***BCL6*** | *CDH11* | *CYLD* | *ELF4* | *FRYL* | *IGF1R* | *LCN2* | *MORC4* | ***NSD2*** | *PLXNA1* | *RFTN1* | *SF1* | *TBX3* | *UGGT2* |
| *ABL1* | *BCL7A* | *CDH23* | *DAP3* | *ELP2* | *FTCD* | *IGLL5* | *LEP* | *MPDZ* | *NXF1* | *PLXNB3* | *RGS4* | *SF3A1* | *TCF12* | *ULK4* |
| *ACAD9* | *BCL9* | *CDH8* | *DAPK1* | *EMC8* | *FUBP1* | *IKBKB* | *LGALS16* | *MPEG1* | *OCEL1* | *PMS1* | *RGS7* | ***SF3B1*** | ***TCF3*** | *UNG* |
| *ACD* | ***BCOR*** | *CDK4* | *DCAF4L2* | ***EP300*** | *FUT9* | *IKZF1* | *LIFR* | *MPL* | *OGDHL* | *PMS2* | *RHBDD1* | ***SGK1*** | *TCL1A* | *USH1C* |
| *ACTB* | ***BCORL1*** | *CDK7* | *DCDC2B* | *EPCAM* | *FYB* | ***IKZF3*** | *LIG1* | *MRGPRF* | *OR10A2* | *POLB* | ***RHOA*** | *SH2B3* | *TCTN2* | *USP11* |
| *ACTG1* | *BCR* | ***CDKN1B*** | *DCHS2* | *EPHA3* | *GATA1* | *IL13RA2* | *LIG3* | *MS4A1* | *OR1S2* | *POLG* | *RIMS2* | *SH2D1A* | *TERF1* | *USP25* |
| *ACTN2* | *BIRC2* | ***CDKN2A*** | *DCLRE1C* | *EPHB2* | *GATA2* | *IL2RB* | *LIG4* | *MSH2* | *OR5H1* | *POP1* | *RIPK1* | *SHC1* | *TERF2* | *VAPA* |
| *ACTN3* | ***BIRC3*** | ***CDKN2B*** | *DCP1B* | *EPOR* | *GLI3* | *IL2RG* | *LMCD1* | *MSH3* | *P2RY8* | *POSTN* | *RIPK2* | *SI* | *TERF2IP* | *VCAN* |
| *ACTN4* | *BLM* | *CDKN2C* | *DDB1* | *ERAP1* | *GLIS3* | *IL6* | *LRIG3* | *MSH6* | *PABPC1* | *POT1* | *RIPK4* | *SIN3A* | ***TET1*** | *VPS13A* |
| *ADCY2* | ***BRAF*** | *CEBPA* | *DDR2* | *ERBB2* | ***GNA13*** | *IL6R* | ***LRP1B*** | *MSL2* | *PABPC5* | *PPIG* | *RNF213* | *SLC13A3* | ***TET2*** | *VWA2* |
| *ADD2* | *BRCA1* | *CHD1* | ***DDX3X*** | *ERBB4* | *GNAS* | *IL6ST* | *LRRK1* | *MSN* | *PALLD* | *PPM1D* | *RNF31* | *SLC16A7* | *TFCP2* | *WAC* |
| *ADGRL2* | *BRCA2* | *CHD2* | *DDX41* | *ERG* | *GNAT1* | *IL7R* | *LRRK2* | ***MTOR*** | *PAMR1* | *PPP1R9A* | *RNF40* | *SLC17A6* | *TGFBR2* | *WAS* |
| *ADGRV1* | *BRCC3* | *CHD3* | *DGKB* | *ESF1* | *GNB1* | *IQCG* | *LRRN3* | *MUC16* | *PARP1* | *PRAME* | *ROBO2* | *SLC18A1* | *TGM7* | *WDR66* |
| *AFAP1L2* | *BRD2* | *CHD8* | *DHCR7* | *ESX1* | *GON4L* | *IRF2BP1* | *LRTM1* | *MUM1* | *PASD1* | ***PRDM1*** | *ROS1* | *SLC29A2* | *THBS1* | *WDR70* |
| *AFF2* | *BRD4* | *CHEK1* | *DHDH* | *ETS1* | *GPR37* | ***IRF4*** | *LTB* | *MYBBP1A* | *PASK* | *PRDM15* | *RP1* | *SLC30A6* | *TIAM1* | *WDR90* |
| *AKT1* | *BRINP2* | *CHEK2* | *DICER1* | ***ETV6*** | *GPR65* | ***IRF8*** | *LTN1* | ***MYC*** | *PAX6* | *PRDM2* | *RP1L1* | *SLC3A2* | *TINF2* | *WHAMM* |
| *ALDH7A1* | *BRIP1* | *CHKB* | ***DIS3*** | *EVI2A* | *GPS2* | *ITK* | *LUZP4* | ***MYD88*** | *PBXIP1* | *PRF1* | *RPA1* | *SLITRK2* | *TLN2* | *WT1* |
| *ALMS1* | ***BTG1*** | *CHMP4C* | *DLC1* | *EWSR1* | *GRB2* | ***ITPKB*** | *LYN* | *MYH11* | *PCBP1* | *PRKCB* | *RPL11* | *SLITRK3* | *TLR2* | *XBP1* |
| *ALPK2* | ***BTG2*** | *CHPF2* | *DLEU2* | *EXOSC6* | *GRIA2* | *ITPR1* | *LYST* | *MYH4* | *PCDHB2* | *PRKD2* | *RPL15* | *SLITRK5* | *TLR4* | *XIAP* |
| *ANK2* | ***BTK*** | *CHST9* | *DLGAP2* | ***EZH2*** | *GRID1* | *ITPR2* | *MAF* | *MYH9* | *PCDHGA12* | *PRKD3* | *RPL26* | *SLITRK6* | *TMSB4X* | ***XPO1*** |
| *ANKRD17* | *CA4* | *CHSY3* | *DMD* | *FAM205A* | *GRIN2A* | ***JAK1*** | *MAFB* | *MYO18A* | *PCLO* | *PRKDC* | *RPL35A* | *SMARCA2* | *TMTC2* | *XPO4* |
| *ANKRD26* | *CACNA1E* | ***CIITA*** | *DMXL1* | ***TENT5C*** | *GTSE1* | *JAK2* | *MAGT1* | *MYO18B* | *PCSK2* | *PROS1* | *RPL5* | ***SMARCA4*** | ***TNFAIP3*** | *XRCC1* |
| *ANKRD44* | *CACNA2D3* | *CLCN6* | *DNAH5* | *FAM47B* | *HDAC6* | ***JAK3*** | *MAML1* | *MYOM2* | *PDCD11* | *PRPF40B* | *RPS10* | *SMC1A* | *TNFRSF13B* | *XRCC5* |
| *AOC2* | *CAD* | *CLGN* | *DNAH9* | *FAM50A* | *HEATR1* | *KANK2* | *MAP1B* | *NAV3* | *PDCD1LG2* | *PSMA1* | *RPS15* | *SMC3* | *TNFRSF13C* | *YLPM1* |
| *APC* | *CAMTA1* | *CMYA5* | *DNAJC6* | *FANCA* | *HEPH* | *KAT6B* | *MAP2* | *NBN* | *PDCL* | *PSMB5* | *RPS17* | *SMYD1* | ***TNFRSF14*** | *YY1AP1* |
| *AQP2* | *CAPN1* | *CNKSR2* | ***DNMT3A*** | *FANCD2* | *HERC1* | *KCNC2* | ***MAP2K1*** | *NBPF1* | *PDGFC* | *PSMB8* | *RPS19* | *SNX29* | *TNFRSF1B* | *ZBTB9* |
| *ARHGAP28* | ***CARD11*** | *COG3* | *DNMT3B* | *FANCG* | *HFE2* | *KCNH2* | *MAP3K14* | ***NCOR1*** | *PDGFRA* | *PSMB9* | *RPS24* | ***SOCS1*** | *TNFRSF21* | *ZCCHC2* |
| *ARHGAP32* | *CARD6* | *COL11A1* | *DNTT* | ***FAS*** | *HIF1A* | *KCNH6* | *MAP7D3* | *NCOR2* | *PDGFRB* | *PSMD1* | *RPS26* | *SOHLH2* | *TNFSF9* | *ZEB1* |
| ***ARID1A*** | *CASP10* | *COL11A2* | *DOCK4* | *FASLG* | *HIST1H1B* | *KCNQ3* | *MAPK1* | *NEB* | *PDLIM3* | *PSMG2* | *RPS29* | *SP140* | *TNRC6B* | *ZFHX3* |
| *ARID1B* | *CBL* | *COL14A1* | *DOCK8* | *FAT1* | *HIST1H1C* | *KDM4C* | *MAX* | *NELL2* | *PDZD7* | ***PTEN*** | *RPS6KA1* | ***SPEN*** | *TOP2A* | *ZFHX4* |
| *ARID2* | *CCM2L* | *COL16A1* | *DPCR1* | *FAT3* | *HIST1H1D* | *KDM5C* | *MBD1* | ***NF1*** | *PER1* | *PTGFRN* | *RPS7* | *SPINK13* | ***TP53*** | *ZFP36L1* |
| ***ARID5B*** | *CCNB1* | *COL19A1* | *DPPA2* | *FAT4* | ***HIST1H1E*** | ***KDM6A*** | *MCRS1* | ***NFKBIA*** | *PHAX* | *PTPN11* | *RRAGC* | *SRF* | *TP53BP1* | *ZMYM3* |
| ***ASXL1*** | ***CCND1*** | *COL4A2* | *DSC1* | *FBXO11* | *HIST1H2AC* | *KDM6B* | *MDC1* | *NFKBIB* | ***PHF6*** | *PTPN14* | *RUBCN* | *SRRM2* | ***TP63*** | *ZNF117* |
| *ATAD2B* | ***CCND3*** | *COL6A3* | *DSCAM* | *FBXO21* | *HIST1H2AG* | *KIAA0355* | *MED12* | *NFKBIE* | *PHLPP1* | *PTPRCAP* | *RUNX1* | *SRSF2* | *TRAF2* | *ZNF296* |
| ***ATM*** | *CCNT1* | *CPA4* | *DSEL* | *FBXO42* | *HIST1H2BC* | *KIAA1671* | *MED23* | *NFKBIZ* | *PIAS2* | *PTPRD* | *RYR1* | *SRSF8* | ***TRAF3*** | *ZNF429* |
| *ATMIN* | *CCR4* | *CRBN* | *DST* | ***FBXW7*** | *HIST1H2BD* | *KIF5B* | ***MEF2B*** | *NHEJ1* | *PIGN* | *PTPRN* | *S1PR1* | *STAG2* | *TRAF3IP1* | *ZNF474* |
| *ATP10B* | *CD14* | ***CREBBP*** | ***DTX1*** | *FES* | *HIST1H2BG* | *KIR2DL4* | *MEF2C* | *NIN* | *PIK3CA* | *RAD21* | *S1PR2* | *STARD8* | *TRIML2* | *ZNF521* |
| *ATP11C* | *CD19* | *CREG2* | *DUSP2* | *FGFR1* | *HIST1H2BK* | *KIR3DL1* | *MEIS1* | *NIPBL* | *PIK3CB* | *RAD50* | *SALL3* | ***STAT3*** | *TRIP12* | *ZNF608* |
| *ATP6AP1* | *CD27* | *CRYBB2* | *DYNC1H1* | *FGFR2* | *HIVEP2* | ***KIT*** | *MET* | *NLRC5* | *PIK3CD* | *RAD51B* | *SAMD9* | ***STAT5B*** | *TRPM6* | *ZNF708* |
| *ATP6V1B2* | *CD274* | *CRYBG3* | *DYRK1A* | *FGFR3* | *HOXB2* | *KLF2* | *MFHAS1* | *NOA1* | *PIK3R1* | *RAD54B* | *SAMHD1* | ***STAT6*** | *TRRAP* | *ZNF85* |
| *ATR* | ***CD28*** | *CSF1R* | ***EBF1*** | *FGFR4* | *HUWE1* | *KLHDC8B* | ***MGA*** | *NOL9* | ***PIM1*** | *RAG2* | *SBDS* | *STK24* | *TSHZ2* | *ZNF90* |
| *ATRX* | *CD40LG* | *CSF3R* | *ECT2L* | *FGL2* | *ICOS* | ***KLHL14*** | *MGAM* | ***NOTCH1*** | *PKD1* | *RARA* | *SCG3* | *STXBP2* | *TTC27* | *ZRSR2* |
| ***B2M*** | ***CD58*** | *CSNK2A1* | *EEF1A1* | *FIGN* | ***ID3*** | ***KLHL6*** | *MIB1* | ***NOTCH2*** | *PKD1L2* | *RASA2* | *SCRIB* | *SUZ12* | *TTN* | *ZZEF1* |
| *BAGE2* | ***CD70*** | *CTCF* | *EEF1E1* | *FLG* | *IDH1* | ***KMT2A*** | *MIR142* | *NPAT* | *PKHD1* | ***RB1*** | *SEC14L3* | *SVIL* | *TUBB* |  |
| *BARD1* | ***CD79A*** | *CTNNA2* | ***EGFR*** | *FLT3* | ***IDH2*** | ***KMT2C*** | *MKI67* | *NPM1* | *PKHD1L1* | *RBM23* | *SENP6* | *SWAP70* | *TYK2* |  |
| *BAZ2A* | ***CD79B*** | *CUL1* | ***EGR1*** | *FLT4* | *IDH3A* | ***KMT2D*** | *MKL1* | *NPTX2* | *PKM* | *RBMXL3* | *SETBP1* | *SYK* | *TYRP1* |  |

**Supplementary Table S2. List of detected SNVs and indels from patients in exploratory group.**

Genes in the green box indicate genes that are not included in the 112 gene targeted MM NGS panel

*: conventional NGS MM panel was performed using total BMA due to failure of CD138+ enrichment process

Abbreviation: PC, Plasma cell; BMA, bone marrow aspirate

**Supplementary Table S3. List of detected SNVs and indels from validation cohort patients by using ultra high depth sequencing targeting 112 genes with error correction algorithm**

| **Patient ID** | **Bone marrow  PC** | **BM and PB** | **BM only** | **PB only** | **%** | **Potential driver mutation** |
| --- | --- | --- | --- | --- | --- | --- |
|  |  | **BM** | **%** | **PB** |  |  |
| S1 | 47.6% | TRAF3 I323fs | 1.18% | TRAF3 I323fs | 5.03% | Yes |
|  |  | TET2 V1521fs | 0.63% | TET2 V1521fs | 0.45% | Yes |
|  |  | MGA N252S | 4.98% | MGA N252S | 8.80% | Yes |
|  |  | DNMT3A I780N | 0.28% | DNMT3A I780N | 0.59% | No |
|  |  | FBXW7 R224X | 0.10% |  |  | Yes |
|  |  | MGA R91X | 0.10% |  |  | No |
|  |  | CIITA W688X | 0.08% |  |  | Yes |
|  |  | SMARCA4 IVS20+2 T>C | 0.06% |  |  | Yes |
|  |  | KMT2C W2423X | 0.04% |  |  | Yes |
|  |  | KMT2C Q2383X | 0.07% |  |  | Yes |
|  |  | BCORL1 Q1076X | 0.07% |  |  | Yes |
| S2 | 25.4% | XPO1 D512Y | 6.20% | XPO1 D512Y | 4.48% | No |
|  |  | TP63 R643X | 0.10% |  |  | Yes |
|  |  |  |  | CREBBP P773L | 1.56% | No |
| S3 | 64.5% | KRAS Q61H | 2.51% | KRAS Q61H | 3.01% | Yes |
|  |  | KRAS Q61L | 4.82% | KRAS Q61L | 3.91% | Yes |
|  |  | NRAS Q61H | 1.75% | NRAS Q61H | 3.03% | Yes |
|  |  | TP53 F385fs | 0.71% | TP53 F385fs | 0.66% | Yes |
|  |  | TET2 Q626X | 0.16% | TET2 Q626X | 0.18% | Yes |
|  |  | TENT5C I155S | 1.77% | TENT5C I155S | 1.95% | No |
|  |  | NOTCH2 A3F | 9.58% | NOTCH2 A3F | 10.43% | No |
|  |  | EZH2 A682T | 0.20% |  |  | Yes |
|  |  | CIITA IVS14-2 A>G | 0.40% |  |  | Yes |
|  |  | KMT2A R1064X | 0.25% |  |  | Yes |
|  |  | EZH2 IVS16+6 G>A | 0.33% |  |  | No |
|  |  | FOXO1 D475V | 0.67% |  |  | No |
|  |  |  |  | KRAS G12D | 0.16% | Yes |
|  |  |  |  | ATM R3008C | 0.28% | Yes |
|  |  |  |  | ATM IVS39+1 G>T | 0.21% | Yes |
|  |  |  |  | TP53 R248Q | 0.56% | Yes |
| S4 | 13.2% | DNMT3A W860R | 1.14% | DNMT3A W860R | 1.18% | No |
|  |  | TP53 R273H | 0.09% |  |  | Yes |
|  |  | JAK1 W363X | 0.07% |  |  | Yes |
|  |  | TET1 Q973X | 0.08% |  |  | Yes |
|  |  | CREBBP P2285fs | 0.05% |  |  | Yes |
|  |  | NCOR1 Q795X | 0.09% |  |  | Yes |
|  |  | DNMT3A Y432X | 0.36% |  |  | Yes |
|  |  | PAX5 V26fs | 0.16% |  |  | Yes |
|  |  | DNMT3A Y735C | 0.93% |  |  | No |
|  |  | KMT2C S1134N | 2.38% |  |  | No |
|  |  |  |  | MAP2K1 G128V | 0.09% | Yes |
|  |  |  |  | ASXL1 G1026X | 0.09% | Yes |
| S5 | 90.0% | ASXL3 L602H | 1.50% | ASXL3 L602H | 11.57% | No |
|  |  | TP63 R618W | 2.66% | TP63 R618W | 21.19% | No |
|  |  | NOTCH2 IVS3-6 delG | 0.08% | NOTCH2 IVS3-6 delG | 0.99% | No |
|  |  | ATM R805X | 0.07% |  |  | yes |
|  |  | DNMT3A F731V | 0.30% |  |  | No |
|  |  | ATM R1882X | 0.13% |  |  | Yes |
|  |  | FBXW7 Q69X | 0.06% |  |  | Yes |
|  |  | TP53 V218A | 0.08% |  |  | Yes |
|  |  |  |  | MGA W795X | 0.18% | Yes |
|  |  |  |  | KMT2A L592V | 0.52% | No |
|  |  |  |  | KMT2C C394Y | 4.99% | No |
|  |  |  |  | NOTCH2 5' UTR mutation | 11.98% | No |
|  |  |  |  | KMT2C IVS6-7 C>T | 3.99% | No |
| S6 | 11.3% | DNMT3A R598X | 0.24% | DNMT3A R598X | 0.49% | Yes |
|  |  | KMT2C R380fs | 2.36% | KMT2C R380fs | 4.07% | Yes |
|  |  | KMT2C L1022F | 7.60% | KMT2C L1022F | 13.82% | No |
|  |  | KMT2C Q873L | 6.46% | KMT2C Q873L | 6.97% | No |
|  |  | ATM W2291X | 0.10% |  |  | Yes |
|  |  | ATM R248X | 0.07% |  |  | Yes |
|  |  | DNMT3A W306X | 0.10% |  |  | Yes |
|  |  | DNMT3A R688H | 0.20% |  |  | Yes |
|  |  | MGA Q1769X | 0.08% |  |  | Yes |
|  |  | JAK1 Q545X | 0.07% |  |  | Yes |
|  |  | CDKN1B Q77X | 0.06% |  |  | Yes |
|  |  | CREBBP W1545X | 0.08% |  |  | Yes |
|  |  | DUSP22 W178X | 0.05% |  |  | Yes |
|  |  |  |  | ASXL1 L950fs | 0.19% | Yes |
|  |  |  |  | SMARCA4 W922X | 0.96% | Yes |
|  |  |  |  | MGA W2410R | 0.27% | No |
|  |  |  |  | MGA K937E | 1.38% | No |
| S7 | 29.4% | TET1 K2076X | 0.35% | TET1 K2076X | 0.62% | Yes |
|  |  | RB1 Q702X | 0.09% |  |  | Yes |
|  |  | RB1 W681X | 0.15% |  |  | Yes |
|  |  | KMT2C S4208N | 0.77% |  |  | No |
|  |  | BCORL1 W1296X | 0.09% |  |  | Yes |
|  |  | TP53 T284A | 0.11% |  |  | Yes |
| S8 | 91.4% | TP53 P34fs | 0.13% | TP53 P34fs | 0.31% | Yes |
|  |  | TET2 IVS7+2T>G | 2.84% | TET2 IVS7+2T>G | 2.30% | Yes |
|  |  | KRAS A146V | 1.48% | KRAS A146V | 1.47% | Yes |
|  |  | TET2 K580fs | 0.55% | TET2 K580fs | 0.40% | Yes |
|  |  | TP53 H178fs | 0.05% |  |  | Yes |
|  |  | TET2 Q720X | 0.12% |  |  | Yes |
|  |  | NRAS Q61R | 0.54% |  |  | Yes |
|  |  | ARID1A P946fs | 0.09% |  |  | Yes |
|  |  | KMT2D V1064fs | 0.11% |  |  | Yes |
|  |  | MGA S492fs | 0.84% |  |  | Yes |
|  |  | STAT5B G472D | 0.13% |  |  | No |
|  |  | MTOR A1823T | 0.15% |  |  | No |
|  |  | KMT2D A5478T | 0.14% |  |  | No |
|  |  | LRP1B K595N | 1.18% |  |  | No |
|  |  | TNFRSF14 M1I | 0.10% |  |  | Yes |
|  |  | DDX3X R603X | 0.12% |  |  | Yes |
|  |  |  |  | TP53 E171X | 0.15% | Yes |
|  |  |  |  | TET3 E760D | 0.33% | Yes |
|  |  |  |  | LRP1B R3638W | 0.37% | No |
|  |  |  |  | DNMT3A IVS17+3A>G | 0.36% | No |

Abbreviation: PC, Plasma cell; BMA, bone marrow aspirate; BM, bone marrow; PB, peripheral blood

**Supplementary Table S4. Application of the Piseq-NGS method in the real laboratory setting: Results of 16 patients tested from March 2022 to July 2023.** NGS testing was initiated using plasma cell-enriched BMAs. In cases where enrichment was unsuccessful, the analysis was conducted on total BMAs.

| **Patient** | **PC enrichment** | **Potential driver mutation** | **Mutation (N)** | **Median VAF (%)** | **Bone marrow PC(%)** | **Aspiration to lab (hr)** |
| --- | --- | --- | --- | --- | --- | --- |
| 1 | Fail | No | 5 | 0.1 | 19.0 | 41.1 |
| 2 | Fail | Yes | 9 | 0.1 | 52.5 | 39.0 |
| 3 | Fail | Yes | 13 | 0.1 | 12.7 | 43.4 |
| 4 | Fail | Yes | 2 | 1.7 | 29.0 | 40.7 |
| 5 | Success | Yes | 5 | 10.4 | 86.6 | 40.0 |
| 6 | Fail | Yes | 4 | 3.7 | 84.8 | 40.0 |
| 7 | Fail | Yes | 12 | 35.2 | 99.4 | 40.1 |
| 8 | Fail | Yes | 4 | 26.3 | 40.3 | 42.5 |
| 9 | Fail | Yes | 8 | 2.5 | 29.3 | 40.3 |
| 10 | Fail | Yes | 12 | 9.1 | 92.4 | 91.8 |
| 11 | Fail | Yes | 5 | 0.4 | 99.6 | 88.5 |
| 12 | Fail | No | 4 | 46.6 | 17.2 | 112.3 |
| 13 | Success | Yes | 6 | 1.9 | 35.2 | 41.9 |
| 14 | Fail | Yes | 7 | 0.5 | 51.5 | 63.5 |
| 15 | Fail | Yes | 11 | 1.4 | 10.8 | 40.1 |
| 16 | Fail | Yes | 7 | 0.5 | 63.3 | 40.9 |

Abbreviation: PC, Plasma cell

**Supplementary Table S5. Comparative analysis of 112 gene mutation frequency.** The mutation prevalence of conventional NGS using plasma cell (PC) are results of 202 myeloma patients tested from June 2017 to February 2022 with 742 gene targeted comprehensive gene NGS panel. The results of PiSeq-MM from validation cohort (n=8) and clinical application in 14 patients, in cases where the analysis was conducted on total BMAs, are compare to larger myeloma cohort studies. Gene with mutations in 22 patients are indicated in bold.

| **Gene** | **Conventional NGS using PC (%) (n=202)** | **Leukemia, 2018 [1] (%, n =418)** | **Cancer Cell, 2014 [2] (%, n=203)** | **MMRF-CoMMpass*  (%, n=995)** | **COSMIC database† [3] (%, n=1229)** | **Validation cohort**  **(n=8)** | **Piseq-MM using BMAs in clinical setting**  **(n=14)** |
| --- | --- | --- | --- | --- | --- | --- | --- |
| ***KRAS*** | 17.8 | 28.9 | 22.0 | 23.7 | 21.0 | BM (2/8) PB (2/8) | BM (4/14) |
| ***NRAS*** | 16.3 | 23.9 | 18.0 | 19.1 | 19.7 | BM (2/8) PB (1/8) | BM (2/14) |
| ***TP53*** | 12.4 | 10.8 | 7.3 | 4.9 | 9.5 | BM (5/8) PB (2/8) |  |
| ***BRAF*** | 9.4 | 9.1 | 6.3 | 7.2 | 6.0 |  | BM (1/14) |
| ***RB1*** | 6.4 | 0.7 | 2.0 | 1.8 | 4.7 | BM (1/8) | BM (3/14) |
| ***TENT5C*** | 4.5 | 11.7 | 7.3 | 12.0 | 10.5 | BM (1/8) PB (1/8) | BM (1/14) |
| ***TRAF3*** | 4.0 | 5.3 | 3.4 | - | 8.9 | BM (1/8) PB (1/8) | BM (2/14) |
| *CCND1* | 3.5 | - | 3.4 | 3.6 | 4.0 |  |  |
| ***DIS3*** | 3.0 | 9.6 | 9.3 | - | 13.5 |  | BM (4/14) |
| ***KMT2D*** | 2.0 | - | 2.0 | 2.3 | 5.6 | BM (1/8) | BM (5/14) |
| ***TET2*** | 2.0 | 4.5 | 0.5 | 2.6 | 4.0 | BM (3/8) PB (3/8) | BM (5/14) |
| ***EP300*** | 1.0 | - | 0.5 | 1.8 | 5.6 |  | BM (1/14) |
| ***ARID1A*** | 1.0 | 3.3 | 1.0 | 1.8 | 3.0 | BM (1/8) | BM (1/14) |
| ***ATM*** | 1.0 | 5.5 | 2.4 | 4.8 | 5.0 | BM (2/8) PB (1/8) | BM (2/14) |
| ***BIRC3*** | 1.0 | 0.2 | - | 2.1 | 3.6 |  | BM (1/14) |
| ***CDKN1B*** | 1.0 | - | 1.5 | 1.7 | 3.0 | BM (1/8) | BM (1/14) |
| *CDKN2B* | 1.0 | - | - | - | - |  |  |
| ***CREBBP*** | 1.0 | 2.4 | 0.5 | 2.5 | 5.6 | BM (2/8) PB (1/8) |  |
| *CXCR4* | 1.0 | - | 1.5 | 1.3 | 1.3 |  |  |
| *NFKBIA* | 1.0 | 1.9 | 0.5 | - | 1.1 |  |  |
| *SF3B1* | 1.0 | 2.6 | 0.5 | 2.0 | 3.4 |  |  |
| ***TNFAIP3*** | 1.0 | - | - | 0.5 | 1.9 |  | BM (1/14) |
| *ARID5B* | 0.5 | 1.7 | 0.5 | - | 6.0 |  |  |
| *CCND3* | 0.5 | 0.5 | - | 1.5 | 1.9 |  |  |
| *CDKN2A* | 0.5 | 0.2 | - | 0.8 | 2.2 |  |  |
| ***DNMT3A*** | 0.5 | 1.4 | - | 1.5 | 7.0 | BM (4/8) PB (4/8) | BM (5/14) |
| *ETV6* | 0.5 | 0.2 | 0.5 | 0.6 | 4.4 |  |  |
| *HIST1H1E* | 0.5 | 2.2 | - | - | 2.5 |  |  |
| ***KIT*** | 0.5 | - | 1.0 | 0.6 | 2.4 |  | BM (1/14) |
| ***KMT2C*** | 0.5 | - | 1.5 | 3.1 | 13.5 | BM (4/8) PB (2/8) |  |
| *MYC* | 0.5 | 1.4 | 1.0 | 1.7 | 3.6 |  |  |
| *MYD88* | 0.5 | 0.2 | 0.5 | 0.7 | 0.1 |  |  |
| ***PRDM1*** | 0.5 | 2.9 | 2.9 | 3.3 | 3.6 |  | BM (1/14) |
| ***PTEN*** | 0.5 | 0.5 | - | 2.5 | 3.1 |  | BM (1/14) |
| *STAT6* | 0.5 | - | - | 0.3 | 1.9 |  |  |
| ***LRP1B*** | - | 8.1 | 5.4 | 7.6 | 12.3 | BM (1/8) PB (1/8) | BM (1/14) |
| *IRF4* | - | 5.5 | 2.0 | 4.1 | 3.8 |  |  |
| ***KMT2A*** | - | - | 1.5 | 3.8 | 5.7 | BM (1/8) PB (1/8) | BM (1/14) |
| ***SETD2*** | - | 4.1 | 0.5 | 3.3 | 5.1 |  | BM (3/14) |
| *BCL11B* | - | - | 0.5 | 3.3 | 3.4 |  |  |
| ***TP63*** | - | - | 0.5 | 3.1 | 6.3 | BM (2/8) PB (1/8) | BM (1/14) |
| *NSD2* | - | - | 2.4 | 3.1 | 6.0 |  |  |
| *SGK1* | - | - | 0.5 | 3.0 | 3.4 |  |  |
| *BTG1* | - | - | 1.5 | 3.0 | 2.8 |  |  |
| ***BIRC6*** | - | - | 0.5 | 2.9 | 10.4 |  | BM(2/14) |
| ***PIM1*** | - | - | 1.5 | 2.9 | 3.0 |  | BM (1/14) |
| ***DDX3X*** | - | 1.7 | 0.5 | 2.8 | 1.9 | BM (1/8) |  |
| ***NF1*** | - | 6.0 | 1.5 | 2.7 | 6.7 |  | BM (1/14) |
| ***PAX5*** | - | - | - | 2.7 | 4.4 | BM (1/8) |  |
| *EGFR* | - | 1.0 | 2.4 | 2.6 | 6.0 |  |  |
| ***UBR5*** | - | - | 1.5 | 2.6 | 4.1 |  | BM (1/14) |
| ***NFKB2*** | - | 1.7 | 0.5 | 2.3 | 1.1 |  | BM (1/14) |
| *TBL1XR1* | - | - | 0.5 | 2.2 | 5.2 |  |  |
| ***NCOR1*** | - | 4.3 | - | 2.1 | 9.3 | BM (1/8) | BM (1/14) |
| ***ASXL1*** | - | 1.9 | - | 1.9 | 3.4 | PB (2/8) |  |
| ***ALK*** | - | 2.2 | 0.5 | 1.7 | 8.0 |  | BM (1/14) |
| ***NOTCH2*** | - | 2.4 | - | 1.7 | 5.8 | BM (2/8) PB (2/8) | BM (1/14) |
| *SOCS1* | - | - | 0.5 | 1.7 | 1.1 |  |  |
| ***SPEN*** | - | 4.8 | 2.0 | 1.6 | 6.3 |  | BM (1/14) |
| ***STAT3*** | - | - | 0.5 | 1.6 | 4.2 |  | BM (1/14) |
| ***CARD11*** | - | - | 2.0 | 1.6 | 4.0 |  | BM (2/14) |
| ***TET1*** | - | - | 0.5 | 1.6 | 3.3 | BM (2/8) PB (1/8) |  |
| ***FOXO1*** | - | - | - | 1.5 | 3.0 | BM (1/8) |  |
| *BCL2* | - | - | - | 1.4 | 5.2 |  |  |
| ***CIITA*** | - | - | - | 1.4 | 1.1 | BM (2/8) |  |
| ***BTK*** | - | 0.7 | - | 1.4 | 0.0 |  | BM (1/14) |
| ***EBF1*** | - | - | 0.5 | 1.3 | 8.2 |  | BM (1/14) |
| ***NOTCH1*** | - | - | 1.0 | 1.3 | 3.3 |  | BM (1/14) |
| *TCF3* | - | - | - | 1.3 | 2.7 |  |  |
| ***MTOR*** | - | - | 0.5 | 1.2 | 6.9 | BM (1/8) | BM (2/14) |
| ***FBXW7*** | - | 2.6 | - | 1.2 | 2.6 | BM (2/8) |  |
| *PHF6* | - | - | 0.5 | 1.2 | 0.8 |  |  |
| ***STAT5B*** | - | - | - | 1.0 | 1.9 | BM (1/8) | BM (1/14) |
| *SETD1B* | - | - | - | 1.0 | 1.5 |  |  |
| ***BCL6*** | - | 0.5 | 0.5 | 1.0 | 0.1 |  | BM (1/14) |
| *KDM6A* | - | 2.2 | - | 0.9 | 3.9 |  |  |
| ***SMARCA4*** | - | - | 0.5 | 0.9 | 3.7 | BM (1/8) PB (1/8) | BM (1/14) |
| ***EZH2*** | - | 1.0 | - | 0.9 | 2.0 | BM (1/8) | BM (1/14) |
| *JAK3* | - | - | - | 0.9 | 1.4 |  |  |
| ***BCOR*** | - | 1.2 | 0.5 | 0.8 | 1.5 |  | BM (2/14) |
| ***TNFRSF14*** | - | - | - | 0.8 | - | BM (1/8) |  |
| ***XPO1*** | - | 1.2 | - | 0.7 | 2.6 | BM (1/8) PB (1/8) |  |
| *IDH2* | - | 1.2 | 0.5 | 0.7 | 0.8 |  |  |
| ***MAP2K1*** | - | - | 0.5 | 0.6 | 2.5 | PB (1/8) |  |
| ***BCORL1*** | - | - | 1.0 | 0.5 | 4.3 | BM (2/8) |  |
| *FAS* | - | - | - | 0.5 | 1.5 |  |  |
| *B2M* | - | - | 1.0 | 0.5 | 0.5 |  |  |
| *REL* | - | - | - | 0.4 | 1.1 |  |  |
| ***JAK1*** | - | - | - | 0.4 | 1.1 | BM (2/8) |  |
| *CD79B* | - | - | - | 0.4 | - |  |  |
| *ID3* | - | - | - | 0.4 | - |  |  |
| *CD28* | - | - | - | 0.3 | 0.4 |  |  |
| *RHOA* | - | - | - | 0.2 | 1.5 |  |  |
| *PLCG1* | - | - | 0.5 | 0.1 | 1.9 |  |  |
| *CD79A* | - | 0.5 | - | 0.1 | 0.7 |  |  |
| ***ASXL3*** | - | - | 1.5 | - | 7.1 | BM (1/8) PB (1/8) | BM (1/14) |
| ***MGA*** | - | 3.3 | 0.5 | - | 7.1 | BM (3/8) PB (3/8) | BM (1/14) |
| ***EGR1*** | - | 2.6 | 3.4 | - | 4.9 |  | BM (4/14) |
| ***KLHL6*** | - | - | 0.5 | - | 3.7 |  | BM (3/14) |
| ***DUSP22*** | - | - | - | - | 3.0 | BM (1/8) |  |
| ***FYN*** | - | - | - | - | 3.0 |  | BM (1/14) |
| ***IKZF3*** | - | 1.9 | 0.5 | - | 2.7 |  | BM (1/14) |
| *BTG2* | - | - | - | - | 2.6 |  |  |
| ***IRF8*** | - | - | 1.0 | - | 2.6 |  | BM(1/14) |
| *KLHL14* | - | - | 0.5 | - | 2.6 |  |  |
| ***ITPKB*** | - | - | - | - | 2.2 |  | BM (1/14) |
| ***DTX1*** | - | 0.5 | 2.0 | - | 2.2 |  | BM (1/14) |
| *GNA13* | - | - | - | - | 1.9 |  |  |
| ***TET3*** | - | - | - | - | 1.5 | PB (1/8) |  |
| *MEF2B* | - | - | - | - | 1.1 |  |  |
| ***CD58*** | - | - | - | - | 0.7 |  | BM (1/14) |
| *CD7* | - | - | - | - | - |  | BM (4/14) |

Abbreviation: PC, Plasma cell; BMA, bone marrow aspirate; BM, bone marrow; PB, peripheral blood

**Reference**

1. Bolli N, Biancon G, Moarii M, Gimondi S, Li Y, de Philippis C, Maura F, Sathiaseelan V, Tai Y-T, Mudie L *et al*: **Analysis of the genomic landscape of multiple myeloma highlights novel prognostic markers and disease subgroups**. *Leukemia* 2018, **32**(12):2604-2616.

2. Lohr JG, Stojanov P, Carter SL, Cruz-Gordillo P, Lawrence MS, Auclair D, Sougnez C, Knoechel B, Gould J, Saksena G *et al*: **Widespread genetic heterogeneity in multiple myeloma: implications for targeted therapy**. *Cancer Cell* 2014, **25**(1):91-101.

3. Forbes SA, Beare D, Boutselakis H, Bamford S, Bindal N, Tate J, Cole CG, Ward S, Dawson E, Ponting L *et al*: **COSMIC: somatic cancer genetics at high-resolution**. *Nucleic Acids Research* 2016, **45**(D1):D777-D783.
